# Principles of MMT

**DOI:** 10.4137/sart.s3742

**Published:** 2009-11-13

**Authors:** Sanju George, Shantala Magdum

**Affiliations:** 1 Consultant and Senior Research Fellow in Addiction Psychiatry, Birmingham and Solihull Mental Health NHS Foundation Trust, UK.; 2 Speciality Doctor in Addiction Psychiatry, Birmingham and Solihull Mental Health NHS Foundation Trust, UK.

We noted with interest the many parallels this study’s (Chen and Fujiwara, Substance Abuse: Research and Treatment 2009:3;61–70) findings have with other similar studies from the western world. In this context, we wish to highlight two crucial principles regarding methadone maintenance treatment (MMT) that ought to guide the clinician. First, given the robust evidence base proving the superiority of MMT plus psychosocial interventions over MMT alone, MMT should always be delivered within a more comprehensive psychosocial treatment program. Second, it is time to shift treatment providers’ emphasis from merely a maintenance treatment/harm reduction perspective to an abstinence/recovery model; these are best viewed as being on a continuum of care and not opposing philosophies. Given the above, our question for the authors is whether theirs was a study that looked at the effectiveness of MMT alone or MMT plus psychosocial interventions. Authors briefly mention patients also having received psychosocial treatments. Further clarity on this would be welcome. Finally, clinicians who work in the west would be interested to know about the formulation, dose range and supervision arrangements of methadone in the study.

Sanju George, MRCPsych

Shantala Magdum, DPM, MSc

